# CUTANEOUS MANIFESTATIONS IN PATIENTS SUSPECTED OF CHIKUNGUNYA DISEASE

**DOI:** 10.4103/0019-5154.53186

**Published:** 2009

**Authors:** Soma Prashant, A S Kumar, D D Mohammed Basheeruddin, T N Chowdhary, B Madhu

**Affiliations:** *From the Department of Dermatology and Venereology, Deccan College of Medical Sciences and Allied Institutes i.e., OwaisiHospital and Princess Esra Hospital, India*

**Keywords:** *Chikungunya disease*, *cutaneous manifestations*, *genital ulcers*

## Abstract

**Context:**

An epidemic of chikungunya disease occurred in India during late 2005 through 2006 affecting nearly 1,400,000 people.

**Aim:**

To study the cutaneous manifestations in suspected cases of chikungunya disease.

**Settings and Design:**

Patients who attended our outpatient departments from January 2006 to September 2006 were prospectively included if they had symptoms of chikungunya disease according to the ‘case definition’ of the National Institute of Communicable Diseases, Directorate General of Health Services, Government of India. The criteria were an acute illness characterized by the sudden onset of fever and several symptoms such as joint pain, headache, backache, photophobia, and eruption during an epidemic of chikungunya fever in the absence of confirmatory serological tests.

**Materials and Methods:**

A total of 115 patients (65 men and 50 women) who satisfied the above criteria were enrolled for the study.

**Results:**

An erythematous maculopapular rash subsiding without any sequelae in 3-4 days was the most common cutaneous finding in our patients. Genital ulcers distributed predominantly over the scrotum and base of the penile shaft in men and labia majora in women were the second most common manifestation. Other manifestations included tenderness/edema of hands and feet, grouped hyperpigmented macules over the nose and cheeks, fixed drug eruptions, erythema nodosum, erythema multiformae, generalized urticarial eruptions, and flare up of pre-existing psoriasis and lichen planus.

**Conclusions:**

To conclude, a plethora of cutaneous manifestations were noted in suspected cases of chikungunya disease. Genital ulcers, to the best of our knowledge, have not been reported during the earlier epidemics but have been reported by others during the present one.

## Introduction

An epidemic of chikungunya disease occurred in India during late 2005 through 2006 affecting nearly 1,400,000 people.[1] We describe the cutaneous manifestations in patients we treated at the outpatient departments of our allied institutes.

Chikungunya virus is an arthropod-borne virus (genus *Alphavirus*, family *Togaviridae*),[2] which was first isolated in Tanzania in 1953.[3] Since then, chikungunya outbreaks have been reported in Africa and Asia.

The last reported case in India was in 1973. Transmission to humans occurs through bites of *Aedes* (mainly *Aedes aegypti* and *A albopictus*) mosquitoes.[4–8]

## Materials and Methods

The patients who attended our outpatient departments from Jan 2006 to Sept 2006 were prospectively included in the study if they fulfilled the criteria for ‘suspect cases’ of chikungunya infection stipulated by the National Institute of Communicable Diseases, Directorate General of Health Services, Government of India.

‘Suspect cases’ have been defined as patients presenting with an acute illness characterized by the sudden onset of fever, with several symptoms such as joint pain, headache, backache, photophobia, and eruption during an epidemic of chikungunya fever and in the absence of confirmatory serological tests.[9] A total of 115 patients (65 men, 50 women) who satisfied the above criteria were enrolled for the study.

## Results

**Figure 1:**
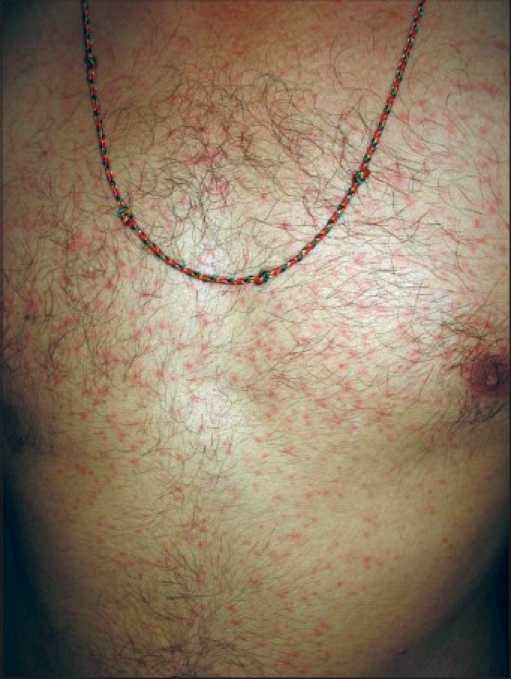
Maculopapular rash on the trunk

**Figure 2:**
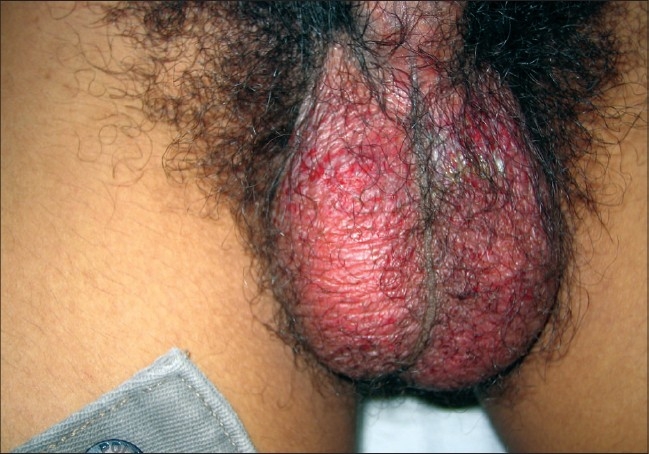
Diffuse erythema of scrotum with erosions

**Figure 3:**
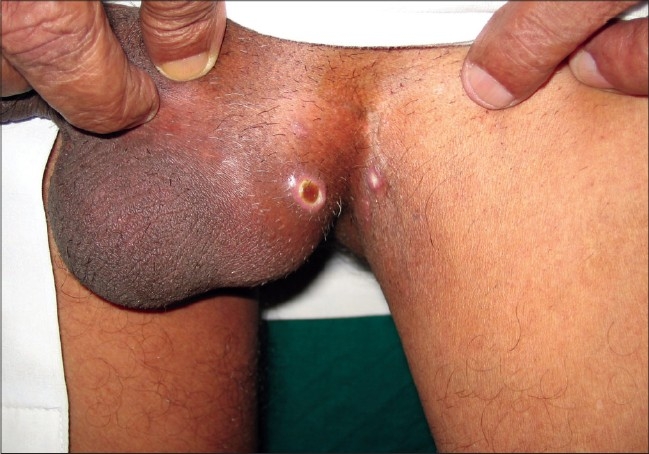
Apposing ulcers on the scrotum and the medial part of thigh

**Figure 4:**
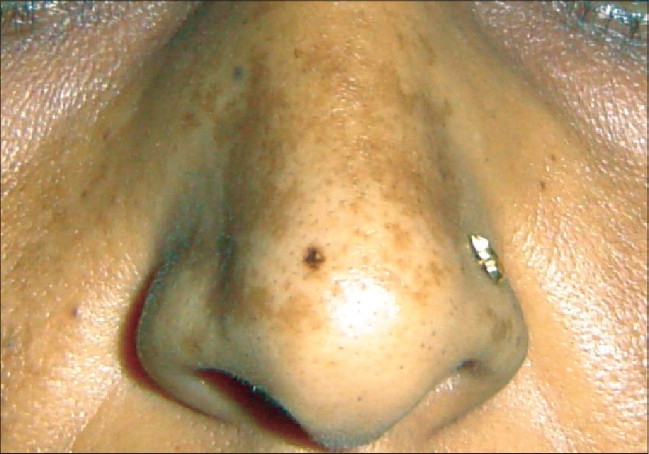
Pigmentation over nose

The most common cutaneous finding was an erythematous maculopapular exanthem seen in 41 patients [Figure 1]. Genital ulcers were seen in 27 (20 men, 7 women) patients [Figures 2 and 3]. Associated bilateral, tender, inguinal lymphadenopathy was seen in seven of the patients. In seven of these patients oral ulcers resembling minor aphthae were seen. Tenderness/edema of hands and feet was observed in 24 patients and in half of these patients it was either concomitant with macular exanthems or with ulcers on the genitalia. Twelve patients showed grouped, hyperpigmented macules coalescing in some areas, distributed predominantly over the nose and cheeks [Figure 4]. Five patients showed discrete, scattered hyperpigmented macules over the face and trunk. The number of lesions ranged from 1-3. Three patients had tender, deep-seated nodules scattered over the distal part of the extremities (erythema nodosum type). Two patients had targetoid lesions over the extremities and trunk simulating an erythema multiform-like eruption. Two patients had generalized urticarial eruptions. Flare up of pre-existing psoriasis and lichen planus was noted in six and four patients respectively. One patient had multiple crusted lesions on the right side of upper lip and angle of mouth [Table 1].

**Table 1:** Type of cutaneous manifestation in chikungunya patients*

Manifestation type	Number of patients affected (%)
Maculopapular rash	41 (35.65)
Genital ulcers	27 (23.47)
Tenderness/edema of hands and feet	24 (20.86)
Centrofacial pigmentation	12 (10.43)
Discrete hyperpigmented macules over the face and trunk	5 (4.34)
Erythema nodosum	3 (2.60)
Targetoid lesions	2 (1.73)
Urticarial rash	2 (1.73)
Flare up of psoriasis	6 (5.21)
Flare up of lichen planus	4 (3.47)
Crusted lesions near angle of mouth	1 (0.86)

* N = 115

## Discussion

Chikungunya fever is a self-limiting viral disease characterized by arthritis mostly involving the wrist, ankle, knee, and small joints of the extremities associated with rashes and fever.[10] The first reported outbreak of Chikungunya fever in India was in 1963. Since then, there have been two more outbreaks, the current one being the fourth.[9] The present outbreak started in late 2005 and continued through 2006 affecting many parts of the country, with southern India reporting the maximum number of cases. The presence of immunoglobulin M (IgM) antibody against the virus has been confirmed in the blood of the affected patients.[11] The virus has been isolated at the National Institute of Virology, Pune, India, and was found to be of the African genotype.[1]

An erythematous maculopapular lesion subsiding in 3-4 days without any sequelae was the most common presenting complaint in the patients we had seen. The rash started with the onset of fever in majority of the patients. In others it started 3-4 days later, usually when the fever was subsiding. Trunk and extremities were the most common areas involved, though a few patients additionally had involvement of face, palms, and soles. The rash was asymptomatic in 80% of the patients, the others complained of mild pruritus. Recurrent crops of maculopapular eruptions occurring probably as a result of successive crops of viremia[12] has been reported, though we did not encounter this in our series of patients. Hochedez *et al*.[13] have reported patients having islands of normal skin on the trunk.

Multiple, painful ulcers with interspersed erythema distributed predominantly over the scrotum in males and labia majora in females was the second most common presentation seen in our OPD and was seen in 27 of our patients. These ulcers started 3-4 days after onset of fever and healed within 1-2 weeks. Some of the ulcers were seen on apposing surfaces of scrotum and thighs or on the ventral surface of penis shaft and scrotum (kissing ulcers). All these patients were screened for HSV-I and -II antibodies (both IgG and IgM). HSV-II IgG antibodies were seen in three of the above patients. IgM antibodies were not seen in any of the patients, denoting a cause other than HSV for the ulcers. Due to paucity of resources VDRL and HIV-ELISA was done in only four patients who had given a history of exposure. The tests were negative in all of them. Pus was sent for Gram staining and for culture and sensitivity in the first seven patients. In five of them no bacteria could be isolated. In one of the patients *Staphylococcal aureus* could be demonstrated, while in the other *Klebsiella sp.* was isolated. The presence of bacteria in only two of the seven patients points toward the fact that the bacteria were secondary contaminants rather than the primary etiological factor. All the patients were treated with broad-spectrum antibiotics and topical mupirocin or fusidic acid. Associated painful, bilateral, nonsuppurative inguinal lymphadenopathy was seen in seven patients. In a recent study published from India by Inamdar *et al*.[14] the authors have reported an incidence of genital ulcers in almost a quarter of their patients, the incidence in our series is almost similar. However, one interesting point is that none of the patients in the study were females, whereas they formed 20% of our series.

Tenderness/edema of palms and soles was noted in 24 patients. In half of them this was concomitant with the macular exanthem or with ulcers on the genitalia. The edema usually lasted for 3-4 weeks.

Twelve patients showed grouped hyperpigmented macules coalescing in some areas distributed predominantly over the nose and cheeks. The lesions started a few days after onset of fever and joint pains and were not associated with preceding erythema. Sunscreens were prescribed to all the patients. The lesions faded in 3-4 weeks without any sequelae. Two other studies done by Inamdar *et al*.[14] and Shivakumar *et al*.[16] have also reported the occurrence of this unusual melanosis.

Five patients showed hyperpigmented macules (no: 1-3) over the face and trunk. The lesions had developed few days after onset of fever and joint pains. History of drug intake was present in all the patients and it consisted of painkillers and antibiotics. There was no prior history of similar lesions in any of the patients. The lesions were suggestive of fixed drug eruption.

Discrete, scattered, deep-seated erythematous nodules over the extremities were seen in three patients. The lesions started a few days after onset of fever. History of drug (antibiotics and pain killers) intake was present in all the cases. The lesions subsided with hyperpigmentation in 1-2 weeks. These lesions were suggestive of erythema nodosum. Exacerbation of pre-existing psoriasis and lichen planus, which were in remission, was also noted.

Studies done by Inamdar *et al*.[14] and Thiruvengadam *et al*.[15] have reported the occurrence of hemorrhagic manifestations in association with chikungunya fever, though in our series of patients we did not encounter similar symptoms.

Flaccid vesiculobullous lesions in infants have also been reported during the present epidemic.[14] These were of sudden onset and generalized and healed without scarring or pigmentary changes.

In the absence of a vaccine against chikungunya virus, avoiding contact with mosquitoes and maintaining good environmental sanitation are the main measures to prevent transmission. Use of insect repellents, mosquito nets, and full-sleeved garments may help minimize exposure to mosquitoes. Vector control measures include the elimination of potential breeding places of mosquitoes. Drums and plastic containers used to store water should be covered. Discarded natural and artificial containers like coconut shells, old tyres, empty bottles, and cans should be properly disposed. Water in air coolers, flower vases, and ornamental water tanks need to be changed regularly. During an outbreak, spraying with 2% pyrethrum in high-risk areas is recommended.[9]

## Conclusions

To conclude, a plethora of cutaneous manifestations were noted in suspected cases of chikungunya. Genital ulcers and the unusual melanosis on the face in chikungunya, to the best of our knowledge, have not been reported during the earlier epidemics, but have been reported by others during the present one.[14] Eruptions like erythema nodosum and erythema multiformae could have been either due to the drugs or due to immunological reaction to the virus itself.
